# Time series analysis of the air pollution around Ploiesti oil refining complex, one of the most polluted regions in Romania

**DOI:** 10.1038/s41598-022-16015-7

**Published:** 2022-07-12

**Authors:** Katalin Bodor, Róbert Szép, Zsolt Bodor

**Affiliations:** 1 Faculty of Natural Sciences, Doctoral School of Chemistry, University of Pécs, Ifjúság 6, 7624 Pécs, Hungary; 2 Department of Bioengineering, Faculty of Economics, Socio - Human Sciences and Engineering, Sapientia Hungarian University of Transylvania, Piaţa Libertăţii 1, 530104 Miercurea Ciuc, Romania; 3 Institute for Research and Development for Hunting and Mountain Resources, str. Progresului 35B, 530240 Miercurea Ciuc, Romania

**Keywords:** Biogeochemistry, Environmental sciences

## Abstract

Refineries and petrochemical industries are known to be the principal sources of emissions for a number of air pollutants, such as Volatile Organic Compounds (VOCs), greenhouse gases and particulate matter, which negatively affect the air quality. The primary goal of this research was the time series analysis of PM_2.5_, PM_10_, As, Cd, Ni, Pb, benzene, toluene, ethylbenzene, o-xylene, m-xylene, p-xylene, CO, NO, NO_2_, NO_x_, SO_2_ and O_3_ over an eleven-year period (2009–2019) and the connection between air pollution and meteorological parameters (air temperature, precipitation quantity and relative humidity). Regarding the pollution level of the major pollutants, the minimum pollution levels, except SO_2_ and O_3_, were recorded during warmer periods, meanwhile increased levels, were detected during the cold period (in winter). The air pollutants’ concentration and distribution are affected by meteorological parameters, such as wind speed and direction, rainfall or even relative humidity. Therefore, the highest concentrations in the winter season were 1.25 times higher than in autumn, 1.3 times higher than the average annual value, 1.57 times higher than in spring and 1.79 times higher than in summer. Monthly variation of O_3_ showed lower concentration during winter (27.62 µg/m^3^) and higher in summer (46.42 µg/m^3^). Based on the statistical analysis, a significant Spearman correlation was detected between the studied air pollutants and meteorological parameters, and according to the Principal Component Analysis (PCA) and cluster analysis, some common sources were also detected.

## Introduction

Air pollution around heavily industrialized regions, especially near the refineries and petrochemical industries, has a negative effect on the environment, therefore causing various health issues in case of the population exposed to ambient air pollution^1,2^. Petrol and oil refineries are the main sources of air, land and water pollutants, hence different emission reduction strategies must be applied to reduce emissions and meet environmental directives^3^. In order to mitigate pollution sources, a number of improvements could be used, for example, CO and CO_2_ capture technology, chemical absorption using monoethanolamine (MEA), physical adsorption and membrane separation^4,5^. In Romania, Ploiești oil-producing and refining is a key area of the economy, and it is neighboring with four large oil refineries^6^. Pollutant emissions are coming from different areas of the petroleum refining: production process, pipelines, flanges, valves, storage tanks and waste zone as well^2^.

A number of toxic and hazardous air pollutants are originated from the petroleum refineries, such as Volatile Organic Compounds (VOCs), particulate matter (PM), trace elements (As, Cd, Ni, Pb), nitrogen oxides (NO, NO_2_) and sulfur dioxide (SO_2_)^7^.

The VOCs released from the petroleum industry include the aliphatic and aromatic hydrocarbons, the most relevant aromatic hydrocarbons are BTEX—(benzene, toluene, ethylbenzene and xylene)^2^. Regarding the exposure, short-term exposure to VOCs may cause several discomforts and morbidity, such as dizziness, fatigue, nausea and depression, while long-term exposure may even result in mutations and different types of cancer^8^. Some of the chemicals emitted from the oil refineries are known or suspected as cancer-causing agents, and on the other hand, are also responsible for developmental and reproductive disorders^9^.

Fine particulate matter (PM_2.5_) with an aerodynamic diameter of up to 2.5 µm, may pass via ingestion, inhalation and dermal absorption causing various health issues, such as respiratory and cardiovascular morbidity and lung cancer^2,10,11^. According to the World Health Organization (WHO) guideline, the PM_10_ and PM_2.5_ annual concentrations must be under 20 µg/m^3^ and 10 µg/m^3^, respectively^12^. Based on the literature, strong correlations were revealed between elevated particulate matter and respiratory and cardiovascular mortality worldwide^13,14^.

The airborne particulate from industrial and traffic emissions may represent an increased health risk since different toxic trace elements and metalloids, such as arsenic, cadmium, molybdenum, nickel, sulfur, selenium, vanadium, zinc and lead are bound to PM^15–17^. Trace elements with important contributions in the development of carcinogenic diseases are Pb, Ni and Cd^18^.

Theozone as a secondary pollutant is formed during the reaction between VOCs andoxides of nitrogen in the presence of heat and sunlight. As a strong oxidizing agent, ozone may induce oxidative damage in the respiratory tract and lungs^19^. Nitrogen oxides may pass through the alveolar-cells (epithelium) and the adjacent capillary vessels of the lungs and destroy the alveolar structures and their function in lungs^20–22^. The petrochemical industry can be the main source of VOCs and NO_x_, which are involved in the ozone formation^23,24^. Several meteorological parameters, such as precipitation quantity, play an important role in pollutant concentration reduction through the washout effect, when air pollutants are washed out by rain, snow or fog^25^.

The main objective of this research study was to analyze the temporal distribution of the selected air pollutants, such as PM_2.5_, PM_10_, As, Cd, Ni, Pb, Benzene (C_6_H_6_), Toluene (C_6_H_5_CH_3_), Ethylbenzene (C_8_H_10_), o-m-p-xylene (C_8_H_10_), CO, NO, NO_2_, NO_x_, SO_2_, and O_3_, and the variation of the meteorological parameters (precipitation quantity, air temperature and relative humidity) over an 11-year period (2009–2019) in Ploiesti, one of the most polluted regions in Romania.

## Materials and methods

### Study area

**Figure 1:**
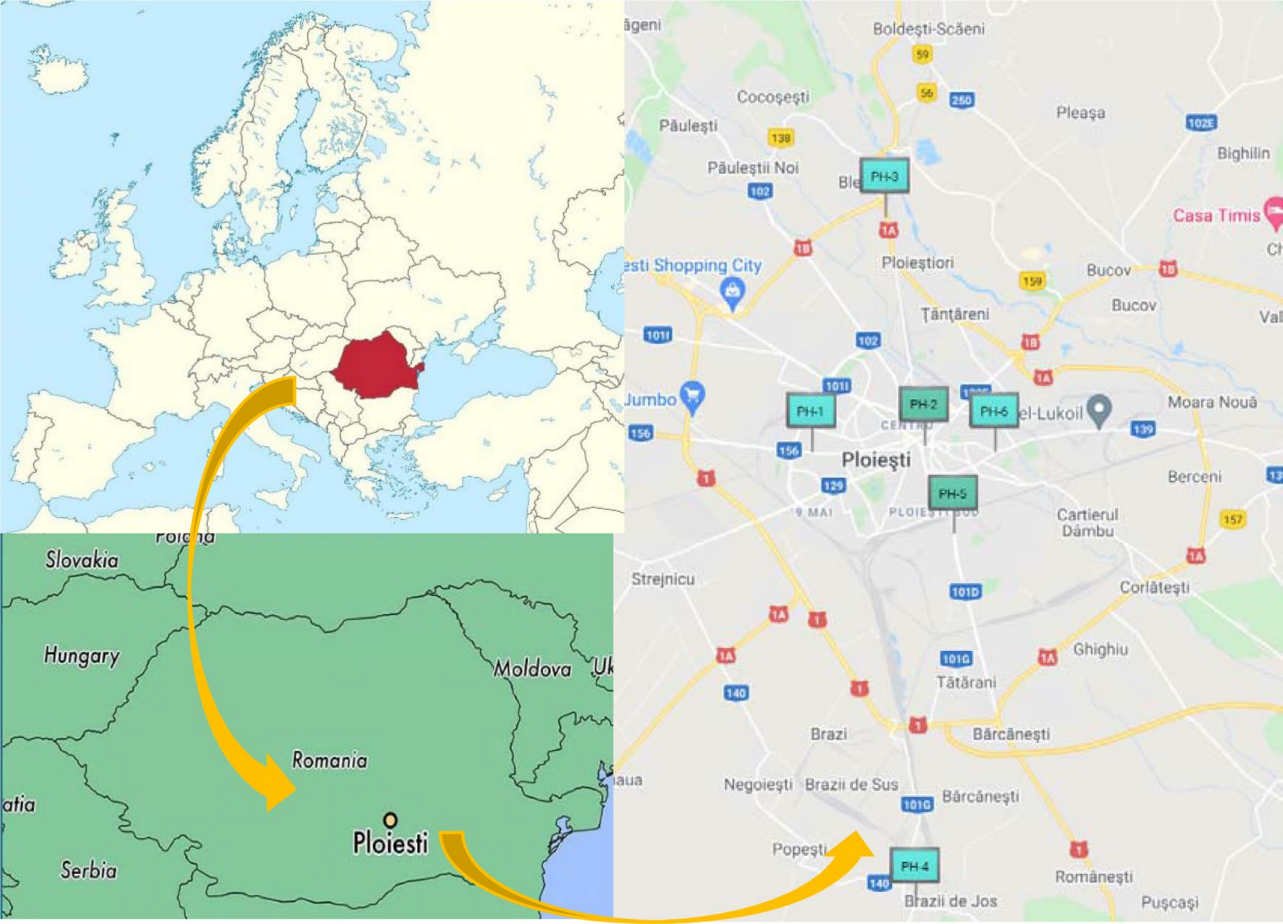
Geographical location of sampling site—Ploiești city^26–28^.

This study was conducted in the Ploiești city which is situated in the south-east of Romania with the following coordinates: latitude: 44° 56′ 24′′ N and longitude: 26° 01′ 24′′ E, at an altitude of 150 m (Fig. 1). The air pollution data were collected from 6 (PH1–PH6) monitoring stations and the average values were used for the statistical analysis. In order to compare the seasonal differences four seasons were defined: winter (December, January, February), spring (March, April, May), summer (June, July, August) and autumn (September, October, November). Ploiești has a temperate humid continental climate, with an average annual temperature of 10.5°C. The oil processing and refining industry is the main economic activity in this region. Regarding the emission of major air pollutants, several industries are present in Ploiești city, hence the emission is mainly related to different industrial activities, such as oil refining, oil extraction machinery and equipment, chemical and manufactured fiber and metalworking facilities as well^6^.

### Pollutants description and statistical methods

The daily data of eleven years (2009–2019) from six monitoring stations (PH1–PH6) were analyzed, and the yearly and monthly variation pattern was deciphered. Air pollutants analyzed are PM_2.5_, PM_10_, As, Cd, Ni, Pb, benzene, toluene, ethylbenzene, o-m-p-xylene, CO, NO, NO_2_, NO_x_, SO_2_ and O_3_. Furthermore, the local meteorological variables were also analyzed in this study by following the precipitation quantity, temperature and relative humidity, respectively. In order to carry out the time series analysis of air pollutants, monthly mean values were calculated from daily concentrations. The daily air pollutant and meteorological data were obtained from the National Air Quality Monitoring Network^29^.

The quantitative analysis of the studied parameters was determined according to the following reference methods: gravimetric method for PM_2.5_ and PM_10_ concentrations^30^; As, Cd, Ni, Pb by inductively coupled plasma-mass-spectrometry (ICP-MS)^31^; benzene by SR EN 14662, 2015^32^; polycyclic aromatic hydrocarbons by SR EN 12884^33^; CO by non-dispersive infrared spectroscopy^34^; NO, NO_2_ and NO_x_ by chemiluminescence^35^; SO_2_ by ultraviolet fluorescence^36^; and O_3_ by ultraviolet photometry^37^.

Annual mean concentrations were calculated by averaging monthly values of each year. For the multiannual trend determination, the Compound Annual Growth Rate (CAGR) was calculated using Microsoft Excel (Eq. (1)).

1 $$CAGR = \left( {\frac{{V_{{final}} }}{{B_{{begin}} }}} \right)^{{\frac{1}{t}}} - 1,$$

where *CAGR*—compound annual growth rate, *V*_*begin*_—beginning value, *V*_*final*_—final value, *t*—time in years.

Descriptive statistic calculations were carried out by using the monthly mean values of the 6 monitoring stations. The multiannual monthly variation of pollutant concentrations was presented using the box plot diagram, where the samples were divided into four quartiles. Spearman correlation analysis was carried out by the widely used *R* (Ri386 3.6.3.) statistical program in order to decipher the relationship between the studied pollutants and meteorological parameters. Furthermore, hierarchical cluster analysis is a commonly used multivariate statistical method in environmental sciences, therefore the classification was done by using the *IBM SPSS Statistics 22* program’s hierarchical cluster analysis method (Ward linkage, Euclidean distance) and the results were visualized in a dendrogram. Principal Component Analysis (PCA) was also implemented^38^, and before PCA analysis, the data were checked using the Keiser–Meyer–Olkin (KMO) test to verify how suited are the air pollution parameters for PCA (Table 1)*.*

**Table 1:** Description of the statistical methods and programs used in this study.

Methods	Programs	Details
Descriptive statistics	Microsoft Excel	Mean, average, standard deviation, 95% confidence interval
Time series analysis	Microsoft Excel	Multiannual concentrations, compound annual growth rate (CAGR)
Box plot analysis	Microsoft Excel	Multiannual monthly mean concentrations
Spearman correlation	R (Ri386 3.6.3.)	Monthly mean concentrations
Hierarchical cluster analysis	IBM SPSS Statistics 22	Monthly mean concentrations
Principal component analysis (PCA)	IBM SPSS Statistics 22	Monthly mean concentrations

## Results and discussions

The main aim of this research was to decipher the relationship between the major air pollutants and meteorological parameters, and to describe the monthly and annually trends around the Ploiești petrochemical industries. According to the statistical analysis of the data recorded at the six automated stations in Ploiești during the studied period was evident that air pollutants with the highest annual average concentration were CO, NO_x_, O_3_, PM_10_ and PM_2.5_ with 0.28 mg/m^3^, 45.97 µg/m^3^, 37.52 µg/m^3^, 30.35 µg/m^3^ and 18.34 µg/m^3^, respectively (Table 2). The petroleum industry requires a huge amount of fossil fuels for operation, and as a consequence the air pollutants release into the atmosphere is significant and in many cases are detrimental to human health^2^. In Ploiești city the multiannual mean PM_10_ and PM_2.5_ concentrations exceed the acceptable limit by 51.75% and 83.4%, respectively. The heavy metal content was determined from the PM_10_ fraction; the rank of metals was Pb > Ni > As > Cd, with 0.73, 0.53, 0.95 and 14.2 ng/m^3^, respectively. The results revealed the most toxic metal (Pb) had a multiannual average of 14.2 ng/m^3^ and mainly originated from the intense oil and petrol refinery activity and heavy traffic. The possible Ni complexes are often salts, nickel oxides, nickel sulfate, nickel sulfide, nickel silicate and nickel chloride^39^.

**Table 2:** Descriptive statistics of the parameters studied from the six automated stations in Ploiești during the study period.

		Min.	25P	Med.	75P	Max.	Avg.	Stdev.
PM_2.5_	[µg/m^3^]	8.54	12.94	16.33	18.91	36.87	18.34	6.45
PM_10_	[µg/m^3^]	15.23	24.26	29.02	27.71	53.75	30.35	7.92
As	[ng/m^3^]	0.19	0.46	0.6	0.43	2.49	0.73	0.45
Cd	[ng/m^3^]	0.06	0.26	0.49	0.19	1.89	0.53	0.34
Ni	[ng/m^3^]	0.03	0.62	0.96	0.59	2.1	0.95	0.41
Pb	[µg/m^3^]	0.0033	0.0106	0.0132	0.0082	0.0401	0.0142	0.0065
Benzene	[µg/m^3^]	0.62	1.85	3.17	4.23	8.25	3.25	1.56
Toluene	[µg/m^3^]	0.26	1.98	3.07	3.35	7.0	3.09	1.36
Ethylbenzene	[µg/m^3^]	0.06	0.38	0.6	0.66	0.84	0.503	0.24
o-xylene	[µg/m^3^]	0	0.12	0.43	0.77	1.77	0.44	0.34
m-xylene	[µg/m^3^]	0.02	0.57	1.05	1.84	2.92	1.07	0.64
p-xylene	[µg/m^3^]	0.01	0.15	0.42	0.83	1.59	0.43	0.3
CO	[mg/m^3^]	0.17	0.24	0.29	0.31	0.41	0.28	0.07
NO	[µg/m^3^]	6.03	8.76	11.15	16.36	32.37	12.28	4.6
NO_2_	[µg/m^3^]	11.51	22.74	26.71	27.21	47.42	27.37	6.82
NO_x_	[µg/m^3^]	21.15	36.48	43.36	52.03	86.74	45.97	12.12
SO_2_	[µg/m^3^]	5	8.77	10.14	8.08	25.52	10.46	2.9
O_3_	[µg/m^3^]	27.82	32.32	35.79	42.09	47.71	37.52	6.54
Prec	[mm]	0.05	1.2	2.41	1.48	23.79	3.22	3.12
Temp	[°C]	-4.02	5.18	12.87	20.54	27.57	13.06	8.73
RH	[%]	50.99	62.61	69.61	81.42	91.32	70.88	9.6

*Min*–minimum, *25P*–25th percentile, *Med*–median, *75P*–75th percentile, *Max*–maximum, *Avg*–average, *Stdev*–standard deviation.

Regarding the organic fraction, the average concentration of benzene determined in the Ploiești region for the studied period (2009–2019) was 3.25 (± 1.56) μg/m^3^, that of toluene 3.09 (± 1.36) μg/m^3^, that of ethylbenzene 0.503 (± 0.24) μg/m^3^ and that of xylene (calculated as the sum of para-, meta- and ortho-xylene) was 1.94 (± 1.28) μg/m^3^. According to the literature, high VOC concentrations were measured near the petrochemical industries around the world^40^.

The highest concentration in case of gaseous pollutants was recorded for CO with 0.28 (± 0.07) mg/L, followed by NO_x_, NO_2_ and NO with 45.97 (± 12.12) μg/m^3^, 27.37 (± 6.82) μg/m^3^ and 12.28 (± 4.6) μg/m^3^, respectively. Analyzing the variation of climatological parameters, the multiannual average daily precipitation quantity was 3.22 (± 3.12) mm, the temperature 13.06°C (± 8.73) and the relative humidity 70.88 (± 9.6).

### Annual variations of pollutants

**Figure 2:**
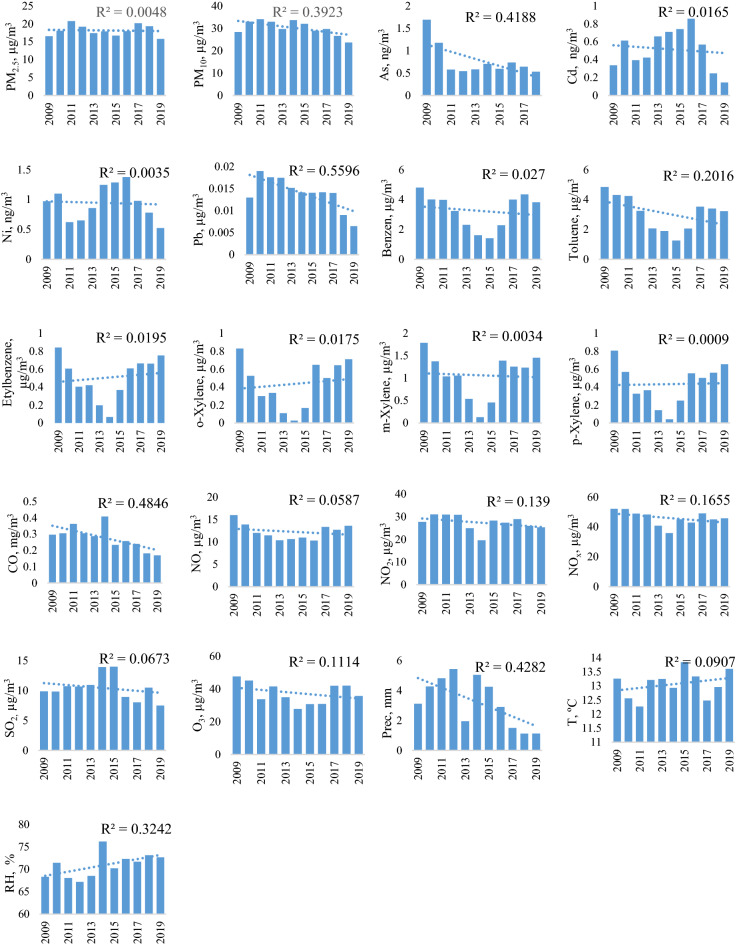
The annual variation of mean concentrations of air pollutants and environmental parameters. Where the dotted lines are trend lines.

The yearly average of air pollutants and climatological parameters are presented in Fig. 2. Compared to the first reference year (2009), except ethylbenzene, o-xylene and temperature, in the last years a decreasing trend can be observed in the annual mean concentrations. However, the annual breakdown shows large variations in case of VOC in 2014 and 2015. An increasing trend was found in case of Cd and Ni between 2012 and 2016 (Fig. 2).

Based on the compound annual growth rate, the highest decreasing trend was observed in the case of As, Cd and Pb with 12.48%, 7.34% and 6.1%, respectively. Between the meteorological parameters the precipitation quantity was reduced by 8.83%, on the other hand, the relative humidity and temperature increased by 0.54% and 0.24%, respectively.

During the studied period, the concentrations of air pollutants decreased continuously due to the implementation of the new ambient air quality standards.

### Monthly multiannual variation of pollutants

**Figure 3:**
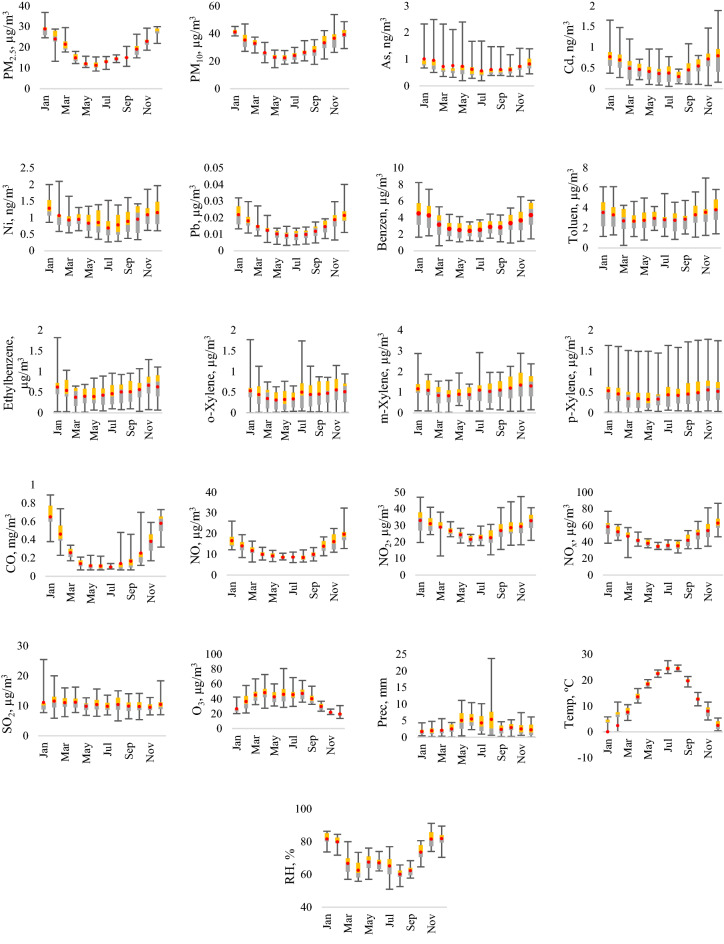
Multiannual monthly variation of the studied air pollutants.

The multiannual monthly variation of the studied parameters in Ploiesti city are presented in Fig. 3. All studied pollutants, except SO_2_ and O_3_, show minimum concentrations during warmer periods, and the maximum concentrations were detected in winter, due to increased emission thanks to the biomass and combustibles consumption in the cold season (heating period). Another important factor is the atmospheric stability during this period, which allows and favors the accumulation of pollutants in the lower atmosphere.

As we expected, the monthly variation of O_3_ showed an opposite trend, lower concentration during winter (27.62 µg/m^3^), and higher concentration in summer (46.42 µg/m^3^). The reduced rate of photochemical formation of O_3_, is in strong correlation with reduced sunshine, lower surface temperature, and higher primary pollutants level. On the other hand, in summer the petrochemical industry emissions of hydrocarbons and NO_x_ are significantly higher, which can result in ozone (O_3_) formation^24^.

The analysis underlines the differences between the seasons: the highest levels of air pollutants were identified in winter season and was 1.25 times higher than in autumn (the minimum ratio of 0.97 was recorded in the case of m-xylene while the maximum of 2.15 for CO), 1.3 times higher than the annual concentration (1.11 as a minimum for m-xylene and 2 as maximum for CO), 1.57 times higher than in spring (the lowest ratio of 1.21 was observed for NO_2−_—meanwhile the highest 3.29 for CO) and 1.79 times higher than in summer (with 1.16 as the minimum for o-xyleneand 4.74 for CO).

Box plots of PM_2.5_, PM_10_, Pb, benzene, toluene, ethylbenzene, o-m-p-xylene, O_3_, NO, NO_2_, NO_x_, SO_2_, (μg/m^3^), CO (mg/m^3^) As, Cd, Ni (ng/m^3^), precipitation (mm), air temperature (°C) and relative humidity (%) multiannual monthly concentrations during the years 2009–2019.

The means are represented by red dots. Box lower (grey) and upper (yellow) limits represent the second and third quartiles. The ends of the whiskers represent the minimum and the maximum values.

The high levels of air pollutants (PM, As, Cd, Ni, Pb, benzene, ethylbenzene, o-m-p-xylene, CO, NO, NO_2_, NO_x_) during the cold season could be mainly associated with meteorological (lower boundary layer, increased atmospheric stability) and environmental factors, for example, emissions from industries, urbanization and motor vehicles^41^.

### Spearman correlation analysis

**Figure 4:**
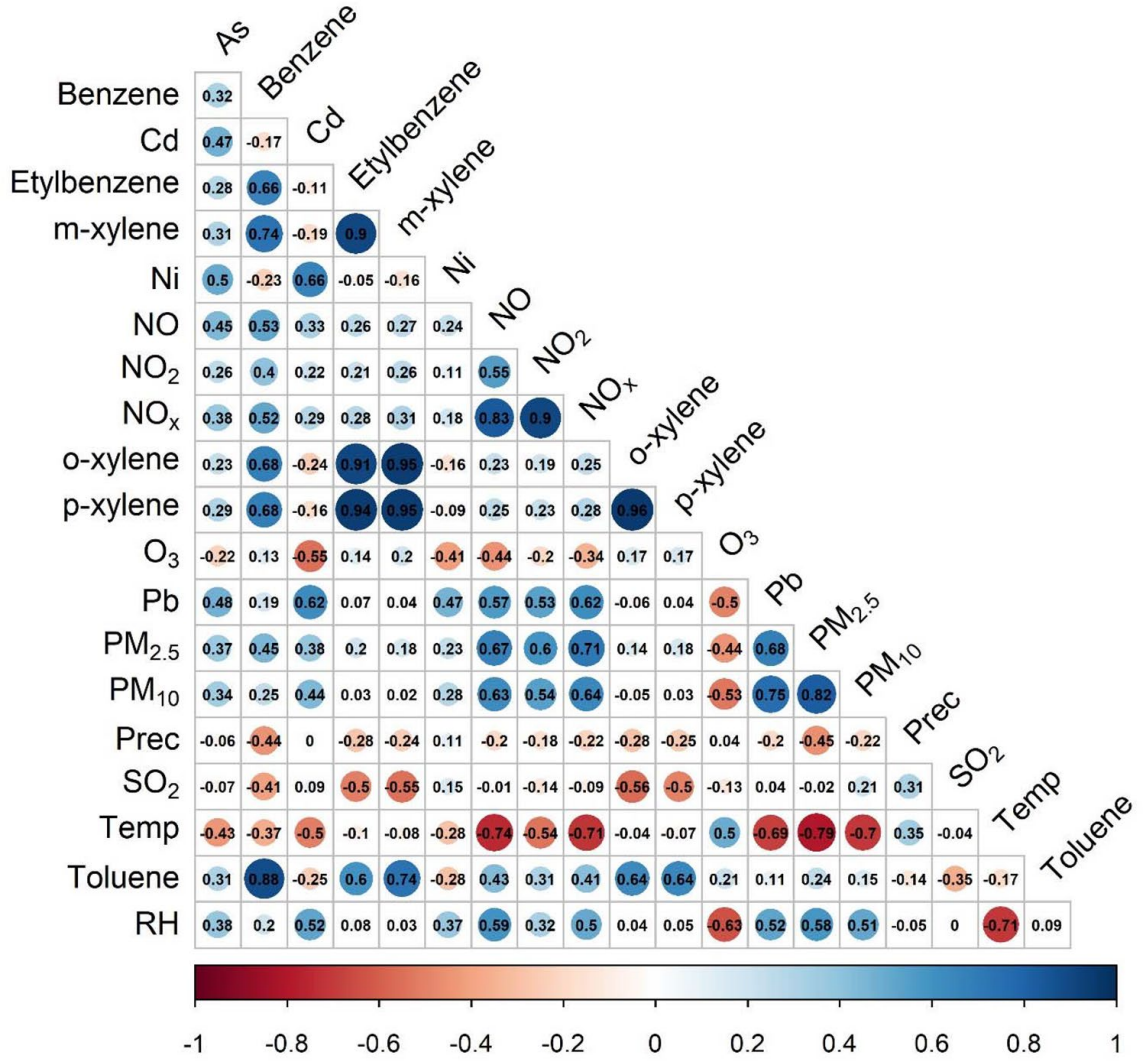
Spearman correlation coefficients between the studied air pollutants and environmental parameters.

To decipher the relationship between the studied parameters, Spearman’s correlation rank analysis was carried out. In total 117 monthly average concentration values were used, and the correlation coefficients between two parameters were considered statistically significant at P < 0.05 and r ≥  + 0.182 and r ≤  − 0.182 (Fig. 4).

Based on the Spearman rank correlation results, a very high positive correlation was observed between the fine (PM_2.5_) and coarse particulate (PM_10_) matter (r = 0.82). All trace elements (As, Cd, Ni and Pb) determined from the PM_10_ fraction show significant correlation of 0.34, 0.44, 0.28 and 0.75, respectively. Furthermore, the BTEX components correlate with each other very well, and the nitrogen oxides show positive correlation with the PM’s, trace elements and BTEX components. Significant negative correlation was found between SO_2_ and BTEX components, and a negative correlation was detected between the O_3_ and all studied parameters, except BTEX.

Furthermore, between PMs and other pollutants, such as CO, NO, NO_2_ and NO_x_ significant strong correlations were identified, except the BTEX compounds where moderate negative correlations were detected between O_3_ and other air pollutants. A moderate correlation was found between SO_2_ and BTEX, r = (− 0.41) − (− 0.56). Regarding the environmental parameters, a strong negative correlation was between air pollutants and environmental parameters, such as temperature and precipitation, while positive correlation was detected with relative humidity. Due to washout effect of precipitation the negative correlation between the precipitation quantity and particulate matters, BTEX and gases is obvious^42^. Further investigations need to be carried out in order to decipher and better understand the underlying processes behind strong and negative correlations as well.

### Hierarchical cluster analysis

**Figure 5:**
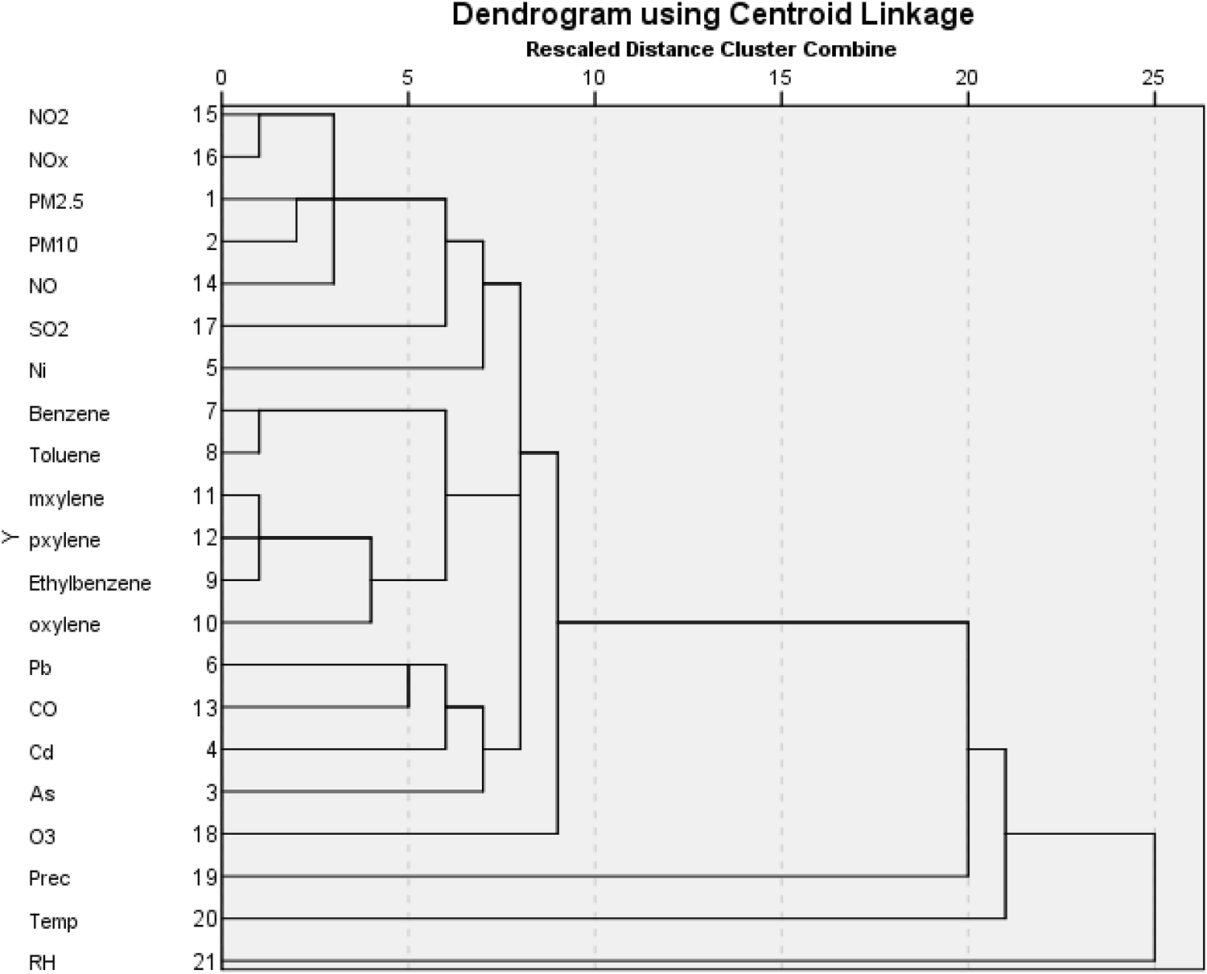
Hierarchical cluster analysis of the studied air pollutants.

The cluster analysis has shown that four main clusters can be identified (Fig. 5). Cluster 1 contains four different sub-clusters: 1.1 contains the PMs and nitrogen oxides, the 1.2 are represented by the trace elements and SO_2_. The 1.3 sub-cluster includes the BTEX components, and in 1.4 sub-cluster is the O_3_. The meteorological parameters—precipitation quantity, air temperature and relative humidity—represent separately the 2, 3 and 4 clusters.

According to the dendrogram, the results suggest that the pollutants share some common sources, hence air pollutants in the same sub-cluster may have common origin^16^. In environmental studies hierarchical clustering analysis methods are the most frequently used approaches to decipher if the pollutants share common sources^43^. In case of CO, NO_2_ and particulate matter emissions, the main responsible sources include the petrol, diesel and other alternative-fuel engines. Furthermore, the BTEX origin are mainly related to the crude oil refinery^44^.

### Principal component analysis (PCA)

**Figure 6:**
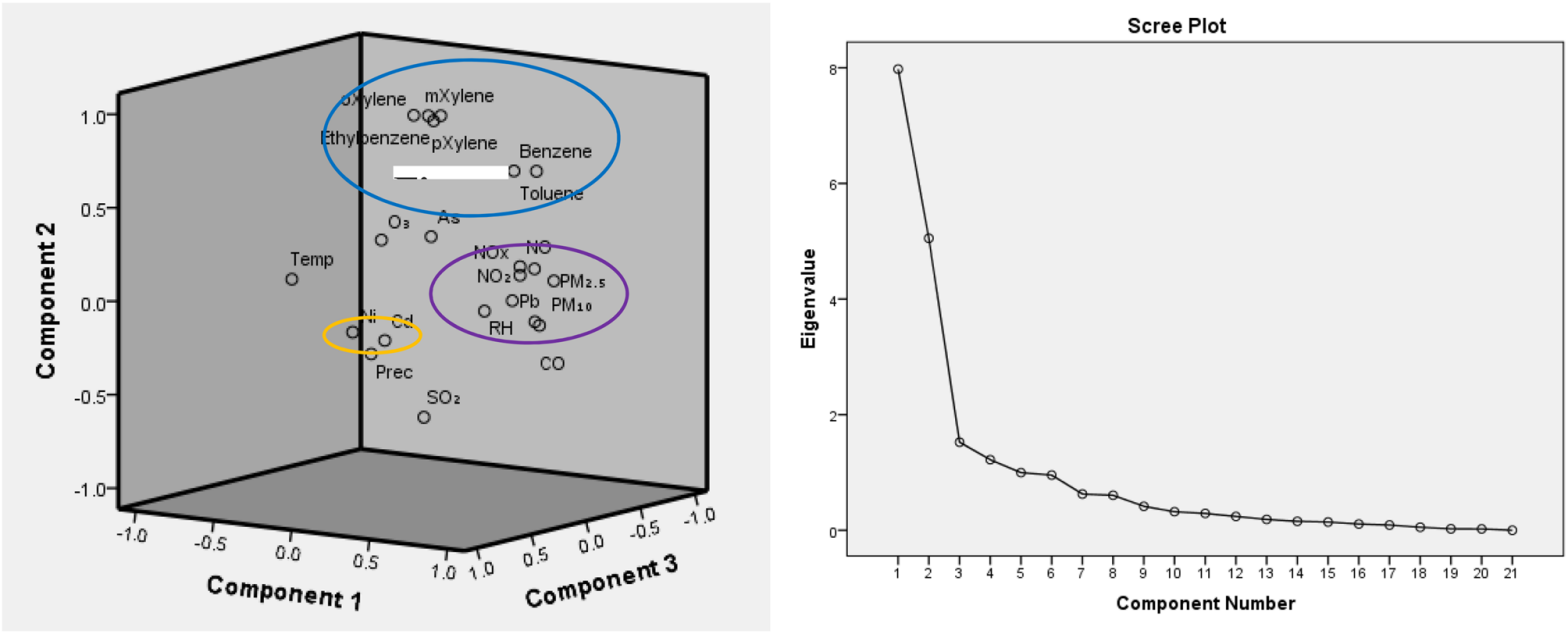
Component plot in rotated space (left), and scree plot (right) of the studied air pollutants.

Principal Component (PC) loadings for all studied air pollutants and for the studied period with corresponding eigenvalues and variances are given in Table 3 and loading factors of air pollutants in three-dimensional spaces are presented in Fig. 6.

**Table 3:** PCA rotated component matrix of the studied air pollutants.

	Component			
	1	2	3	4
PM_2.5_	**0.906**	0.153	0.000	− 0.117
CO	**0.892**	− 0.046	0.154	0.034
Temp.	− 0.882	− 0.042	− 0.151	**0.049**
PM_10_	**0.864**	− 0.080	0.072	− 0.003
NO_x_	**0.838**	0.221	0.081	0.081
NO	**0.785**	0.236	0.137	0.118
Pb	**0.775**	0.061	0.193	0.076
RH	**0.695**	0.018	0.336	− 0.070
NO_2_	**0.689**	0.160	0.001	0.027
O_3_	− 0.507	**0.165**	− 0.437	0.414
p-Xylene	0.091	**0.951**	− 0.012	0.139
o-Xylene	− 0.013	**0.941**	− 0.028	− 0.017
m-Xylene	0.098	**0.937**	− 0.117	0.109
Ethylbenzene	0.128	**0.928**	− 0.008	0.061
SO_2_	0.083	− 0.658	0.017	0.3**20**
Benzene	0.527	**0.645**	− 0.382	0.195
Toluene	0.349	**0.622**	− 0.430	0.393
Ni	0.220	− 0.067	**0.864**	0.113
Cd	0.379	− 0.104	**0.797**	0.033
As	0.247	0.349	0.184	**0.712**
Prec	− 0.276	− 0.360	− 0.013	**0.499**
Eigenvalue	7.977	5.051	1.523	1.22
% variance	33.54	24.89	10.38	6.287
Cumulative % variance	33.54	58.44	68.81	75.1

Significant values are in bold.

Through the PCA the data were transformed into the new coordinate system, the greatest variance by scalar projection is the first component, the second greatest variance is defined as the second component, and the same order is following in the third and the fourth component. According to the Principal Component Analysis, four components were extracted from the component matrix, accounting for 75.1% of the overall variance. Factor 1 contains PM_2.5_, CO, PM_10_, NO_x_, NO, Pb, NO_2_ and RH, and represent 33.54% of the total variance. The factor 2 was represented by O_3_, o-m-p-xylene, ethylbenzene, benzene and toluene. The third and fourth components include Ni and Cd as well as temperature, SO_2_, As and precipitation. The adequacy of the Kaiser–Meyer–Olkin (KMO) measure of sampling was 0.795, followed by the execution of the PCA, which means that the tested samples show good adequacy.

High loadings for PM_2.5_, CO, PM_10_, NO_x_, NO, Pb, RH and NO_2_ in PC_1_ (33.54) suggest that these are the major sources of pollutants in this area. The PC_2_ (24.89%) are represented by the following air pollutants parameters: O_3_, o-xylene, m-xylene, p-xylene, ethylbenzene, benzene and toluene. The PC_3_ with 10.38% total variance shows highest loadings for Cd and Ni. According to Pandey et al. Cd and Ni have significant contributions from crude–oil combustion and metallurgical industrial areas and motor vehicle emissions^45^. As shown in Fig. 6, the studied parameters which are localized near to each other are in the same cluster.

## Limitation

Greenhouse gases (CH_4_, N_2_O, CO_2_) were not studied in this article because they were not monitored during the studied period.

## Conclusions

The aim of the present study was to identify the level air pollution and the relationship between environmental parameters and pollutants in one of the most polluted regions in Romania, in Ploiești, during the studied period (2009–2019). The results revealed that the application of time series analysis can give information on the temporal trends of pollutants near the oil refinery. In concordance with many other industrial activities, petrochemical industry and refineries have a negative effect on air quality. Compared to the first reference year (2009), except ethylbenzene, o-xylene and temperature, all studied air pollutants showed a decreasing trend during the studied years. Seasonal analysis of the pollutant concentrations revealed that the highest values were measured in cold season, especially in winter. Variation of O_3_ and SO_2_ showed an opposite trend, lower concentration in winter and higher concentration in summer. Based on the cluster analysis some pollutants may share common sources, air pollutants in the same sub-cluster may have common origin.

In order to minimize the human health effects of the air pollutants it is essential to respect the Air Quality standards, especially near refineries.

The statistical interpretation of the air pollution distribution can contribute to the development of new and more sector-specific and regional air quality standards regarding oil refineries to reduce air pollution.

The main reason why pollution levels decreased in the last years was mainly attributed to the implementation of the new air quality standards for the industry and on the other hand to the changes in transportation, more sustainable and eco-friendly.

## Data Availability

The datasets used and analyzed during the current study are available from the corresponding author on reasonable request.
