# Author Correction: Time series analysis of the air pollution around Ploiesti oil refining complex, one of the most polluted regions in Romania

**DOI:** 10.1038/s41598-022-17284-y

**Published:** 2022-07-26

**Authors:** Katalin Bodor, Róbert Szép, Zsolt Bodor

**Affiliations:** 1 Faculty of Natural Sciences, Doctoral School of Chemistry, University of Pécs, Ifúság 6, 7624 Pécs, Hungary; 2 Department of Bioengineering, Faculty of Economics, Socio - Human Sciences and Engineering, Sapientia Hungarian University of Transylvania, Piaţa Libertăţii 1, 530104 Miercurea Ciuc, Romania; 3 Institute for Research and Development for Hunting and Mountain Resources, str. Progresului 35B, 530240 Miercurea Ciuc, Romania

Correction to: *Scientific Reports* 10.1038/s41598-022-16015-7, published online 12 July 2022

In the original version of this Article, Róbert Szép was incorrectly affiliated with ‘Faculty of Natural Sciences, Doctoral School of Chemistry, University of Pécs, Ifúság 6, 7624 Pécs, Hungary’.

The correct affiliations are listed below.

Department of Bioengineering, Faculty of Economics, Socio—Human Sciences and Engineering, Sapientia Hungarian University of Transylvania, Piaţa Libertăţii 1, 530104 Miercurea Ciuc, Romania.

Institute for Research and Development for Hunting and Mountain Resources, str. Progresului 35B, 530240 Miercurea Ciuc, Romania.

The original Article has been corrected.

